# Consortium inoculum of five thermo-tolerant phosphate solubilizing Actinomycetes for multipurpose biofertilizer preparation

**Published:** 2017-10

**Authors:** Arusha P. Nandimath, Dilip D. Karad, Shantikumar G. Gupta, Arun S. Kharat

**Affiliations:** 1 Department of Biotechnology, Dr. Babasaheb Ambedkar Marathwada University, Subcampus, Osmanabad, MS 413501, Maharashtra, India; 2 Department of Microbiology, Shri Shivaji Mahavidyalaya, MS 413411, Barshi, Maharashtra, India; 3 Government Institute of Forensic Sciences, MS 431004, Aurangabad, Maharashtra, India

**Keywords:** Phosphate solubilizing actinomycetes, Thermo-tolerant, Consortium inoculum, Biofertilizer

## Abstract

**Background and Objectives:**

Alkaline pH of the soil facilitates the conversion of phosphate present in phosphate fertilizer applied in the field to insoluble phosphate which is not available to plants. Problem of soluble phosphate deficiency arises, primarily due to needless use of phosphate fertilizer. We sought to biofertilizer with the thermo-tolerant phosphate solubilizing actinomycetes consortium that could convert insoluble phosphate to soluble phosphate at wider temperature range.

**Materials and Methods:**

In the present investigation consortium of five thermo-tolerant phosphate solubilizing actinomycetes was applied for preparation of inoculum to produce multipurpose bio-fertilizer. Phosphates solubilizing thermo-tolerant 32 actinomycetes strains were processed for identification with the use of PIBWIN software and were screened for phosphate solubilizing activity.

**Results:**

Amongst these five actinomycetes were selected on the basis of their ability to produce cellulase, chitinase, pectinase, protease, lipase, amylase and phosphate solubilizing enzymes. Ability to produce these enzymes at 28°C and 50°C were examined. Biofertilizer was prepared by using agricultural waste as a raw material. While preparation of bio-fertilizer the pH decreased from 7.5 to 4.3 and temperature increased up to 74°C maximum at the end of 4^th^ week and in subsequent week it started to decline gradually till it reached around 50°C, which was found to be stable up to eighth week. This thermo-tolerant actinomycetes consortium released soluble phosphate of up to 46.7 μg ml^−1^.

**Conclusion:**

As the mesophilic organisms die out at high temperature of composting hence thormo-tolerant actinomycetes would be the better substitute for preparation of phosphate solubilizing bio-fertilizer with added potential to degrade complex macromolecules in composting.

## INTRODUCTION

Solubilization of insoluble phosphorous by microorganisms was reported by Pikovskaya (1). During the last 25 years knowledge on phosphate solubilizing microorganisms (PSM) has increased significantly (2, 3). Phosphorus is one of the most important nutrients for plant growth. Unfortunately, added phosphorus to the soil as a phosphate fertilizer becomes insoluble immediately due to alkaline pH of the soil, and not available for the plants (4). The soluble phosphorus in phosphate fertilizers is easily and rapidly precipitated to insoluble forms with soil cations or adsorbed to calcium carbonate, aluminum oxide, iron oxide, and aluminum silicate, depending on the particular properties of the soil (5). This process decreases the efficiency with which soluble phosphorus can be taken up by the plants and in turn decreases the effectiveness of fertilizer. This has caused more applications of phosphate fertilizers to agricultural fields. The overuse of phosphate fertilizers has increased agricultural costs and a variety of environmental problems (5). Therefore, the concept of adding PSM as biofertilizer would be economical and ergonomic approach.

The PSM play basic roles in phosphorus cycling in natural and agricultural ecosystems. PSM can change the insoluble phosphorus to soluble forms by polymeric substances formation, exchange reactions, chelation and acidification (6). The principal mechanism for mineral phosphate solubilization is the production of organic acids, and acid phosphatases play a major role in the mineralization of organic phosphorous in soil. Therefore, the use of PSM in agricultural practice would help to reduce high of phosphate solubilization and also mobilize insoluble phosphorus into the fertilizers in the soils to which they are applied. The application of PSM has been shown to release soluble phosphorus, promote plant growth, and protect plants from pathogen infection (7). The PSM have been introduced to farming community as a phosphate biofertilizer. All PSM studied and applied to date have been mesophiles that could only be used under mesophilic conditions. These types of microbes are not appropriate for biofertilizer preparation at the high temperatures (over 50°C) that occur during the first stage of composting (8, 9).

Composting is a natural way of recycling and degrades organic waste into a valuable biofertilizer. Microbes in the biofertilizers that survive compost preparation can tolerate to wide temperature variations, and have various enzyme activities responsible for decomposing complex organic wastes. Strains from the genera *Pseudomonas*, *Bacillus* and *Rhizobium* are among the most powerful phosphate solubilizers studied so far (10, 11).

The objective of the present investigation was to use actinomycete consortium as PSM in biofertilizer preparation and to make the biofertilizer multifunctional with ability to degrade all types of wastes like cellulose, carbohydrate, lipids, chitin and pectin, main macromolecular composition of agricultural waste.

## MATERIALS AND METHODS

### Screening and isolation of phosphate solubilizing actinomycetes.

The compost samples were collected from the villages around Barshi Dist-Solapur, MS, India and enrichment of compost samples were carried out in glycerol asparagine broth supplemented with cycloheximede (80 μg/ml). A 10-fold serial dilutions of the sample were prepared up to 10^−6^ and 0.1 ml aliquots of 10^−5^ and 10^−6^ dilution was inoculated into glycerol asparagine agar (L-asparagine-0.1g, K_2_HPO_4_ - 0.1 g, glycerol-1 g, trace salt solution-0.1 ml, agar-2.5 g, distilled water-100 ml pH-7.4). To avoid the growth of fungal contaminant, medium was supplemented with cycloheximide (80 μg/ml). The glycerol asparagin agar plates were then incubated at 50°C and monitored periodically over 5 to 7 days. Pure isolates were preserved as spore suspension in glycerol for further studies.

Potential phosphate solubilizing actinomycete isolates was screened for their ability to solubilise calcium phosphate present in the Pikovskaya medium. The composition of modified Pikovskaya agar was 10 g glucose, 0.5 g yeast extract, 0.5 g (NH4)_2_SO_4_, 0.2 g KCl, 0.1 g MgSO_4_.7H_2_O, 0.0001 g MnSO_4_.H_2_O, 0.0001 g FeSO_4_.7H_2_O and 20 g agar (1, 12). Tricalcium phosphate (5 g L^−1^) was added to the medium as a sole phosphate source for selectively screening of actinomycetes. A loopful of pure culture was placed on the centre of the same agar plates and incubated for 50°C for 5 days. After 5 days, serial dilution of enriched medium was carried out and aliquots of diluted compost samples were subjected to spread on sterile Pikovskaya’s agar medium. The plates were kept for incubation at 50°C for 6 to 8 days. The phosphate solubilization index of actinomyces was calculated as clear zone / colony size in mm. Actinomycete colonies were purified by picking up spores from single colony. Each actinomycete culture was maintained at 4°C as glycerol suspension.

### Identification of phosphate solubilizing actinomycetes.

For the identification of actinomycetes morphological characteristics were studied with cover slip culture technique. Cultural characteristics were recorded on glycerol asparagine agar medium. Biochemical characters were recorded on the basis of sugar utilization potential, enzymatic activities and growth under inhibitory substances. On the basis of spore mass colour, the substrate mycelium colour, the shape of the spore chain, morphological and cultural characteristics the isolate were tentatively identified as *Streptomyces*. Biochemical characterisations of phosphate solubilizing *Streptomyces* were carried out (13, 14).

Identification of actinomycetes up to the genus level thus was carried out on the basis of microscopic/morphological and cultural characteristics. The species level identification was achieved by studying biochemical and other characters. The members of *Streptomyces* and *Streptoverticillium* genus were identified by using PIBWIN software Version: 2.0 for Windows (15). The PIBWIN software is based on probabilistic identification. Probabilistic identification matrices used were Streptomyces species major cluster (13, 16) and *Streptomyces* species minor cluster (17, 18) and for *Streptoverticillium* species (16).

### Enzyme activity indices thermo-tolerant actinomycetes.

Enzyme activity was measured by plate and broth assays at 28°C and 50°C for 5 and 10 days, respectively. The cellulase activity was determined by Mandels–Reese medium with carboxy-methyl-cellulose (CMC) as the sole carbon source (19, 20). Chitinase and pectinase activities were determined in Mandels–Reese agar with chitin and pectin as the sole carbon source, respectively (21). The α-amylase activity was measured by soluble starch-yeast extract agar as described by Kammoun et al. (22). The lipase activity was measured with tributyrin agar (19). The protease activity was assayed by skim milk agar (23). Ratios of clear zone / colony size (CZ/CS) were calculated and were treated as the enzyme activity indices.

### Determination of phosphate solubilizing activity.

Phosphate solubilizing activity was measured by plate and broth assay at 28°C and 50°C, respectively. Pikovskaya’s medium (PVK) was used to measure calcium phosphate [Ca_3_(PO_4_)_2_] solubilizing activity, while PVK supplemented with 5 g Rock phosphate for RP-solubilization (24). Plate assay were performed for 5 days, and the PSAI-phosphate solubilizing activity index (PSAI = CZ/CS) was calculated (25). Broth assays wee performed for 5 days. The pH and soluble phosphorus in the super-natant were determined with a pH meter and by the colorimetric molybdate blue method, respectively (25, 26).

### Quantitative estimation of soluble phosphate.

The compost samples were prepared by inoculating 1.0 g of each inoculum into 100 ml of sterile distilled water. Homogenization of compost was carried out by keeping it on shaker for 30 minutes at 120 rpm at room temperature. After 30 minutes, samples were removed aseptically and centrifuged at 2,000 rpm for 10 minutes. About 10 ml supernatant was inoculated into 100 ml of sterile Pikovskaya’s broth and kept it at 120 rpm at 50°C for 5 days for enrichment.

The isolates showing zone of solubilization on Pikovskaya’s agar were further examined for their ability to release phosphate in the broth media. Thus, 1.0 ml (O.D 600, 1.0) of each isolates culture (18 hrs old) was inoculated separately into 100 ml of sterile Pikovskaya’s broth in the 250 ml Erlenmeyer flasks. Each flask was incubated at 50°C at 120 rpm for 5 days on rotary shaker. Simultaneously, the uninoculated control was also kept under similar conditions. All the experiments were carried out in triplicate. To estimate the amount of phosphate released by these isolates, 10 ml of each sample was withdrawn at regular intervals of 24 hrs and was examined for soluble phosphorus and pH change. The cultures were harvested by centrifugation at 5,000 rpm for 15 minutes. The soluble phosphorus in the supernatant was measured by chlorostannous reduced molybdophosphoric acid blue method (27). For that, to a 100 μl aliquot of supernatant, 10 ml of chloromolybdic reagent was added and diluted up to 40 ml with distilled water. Added with 5 drops of chlorostannous acid reagent and mixed it by making final volume to 50 ml with distilled water. The absorbance of the resultant colour was recorded at 600 nm against blank in UV visible spectrophotometer and the pH of the supernatant was measured by pH electrode.

While composting with actinomycete consortium, the total soluble phosphorous was extracted by using extraction solution (0.025N HCl and 0.03 N NH_4_F) by modified colorimetric molybdate blue method following Kjeldahl digestion (28).

### Biofertilizer preparation.

The agriculture and animal waste was collected from villages around Barshi town. The waste were crushed into the granules roughly below 50.00 mm in diameter and contained 50% sugar cane bagasse and press mud, 15% fruit and vegetable scraps, 15% slurry from biogas plant, 5% cow dung, 5% slaughter house waste, and 10% sewage waste. The material had pH 7.6 ± 0.2, moisture content 52.5 ± 1.5%, total organic carbon 45.4 ± 1.1%, total nitrogen 3.2 ± 0.5%, carbon to nitrogen (C/N) ratio 14.19 ± 3.1%.

Each gram of dry raw material was inoculated with tested actinomycetes at about 1×10^5^ spores in an initial moisture content of 60–65% in a 100 1itre capacity waterproof plastic drum (60 cm inner diameter and 100 cm height). The compost was turned over every three to four days for 8 weeks. Non-inoculated raw material was used as the control (8).

### Statistical analysis of data.

All of the experiments were carried out in triplicate. The data reported are as means ± SD (standard deviation). The SPSS version 10 for Windows and MS-EXCEL was used for the data analysis.

## RESULTS

### Screening and isolation of phosphate solubilizing actinomycetes.

Actinomycete isolates from compost samples were collected from compost pit compost. Thirty two phosphate solubilizing isolates were selected (Table 1). Each of the isolates was purified on glycerol asparagine agar and maintained at 4°C. On the basis of zone of solubilization in Pikovskaya’s agar medium, total 5 actinomycete strains were screened and selected for further studies (Table 2).

**Table 1.** Screening and identification of phosphate solubilising Streptomyces

**Sr. No.**	**Isolate No.**	**Identification of Actinomycetes**	**PIBWIN ID Score**	**Phosphatase at 28°C**	**Phosphatase at 50°C**
1	AN-1	*Streptomyces californicus*	0.97068	1.35 ± 0.04	1.88 ± 0.05
2	AN-2	*Streptomyces exfoliatus*	1.00000	1.22 ± 0.03	1.49 ± 0.03
3	AN-3	*Streptomyces rimosus*	0.99934	1.13 ± 0.06	1.66 ± 0.01
4	AN-4	*Streptomyces fulvissimus*	0.99881	1.38 ± 0.01	1.53 ± 0.02
5	AN-5	*Streptomyces lydicus*	0.99503	1.45 ± 0.00	1.37 ± 0.04
6	AN-6	*Streptomyces lydicus*	0.99503	1.52 ± 0.03	1.99 ± 0.01
7	AN-7	*Streptomyces chromogenus*	0.99927	1.19 ± 0.08	1.48 ± 0.01
8	AN-8	*Streptomyces fulvissimus*	0.99652	1.24 ± 0.03	1.77 ± 0.02
9	AN-9	*Streptomyces rimosus*	0.99934	1.36 ± 0.05	1.89 ± 0.00
10	AN-10	*Streptomyces violaceus*	0.99670	1.17 ± 0.07	1.35 ± 0.01
11	AN-11	*Streptomyces filipinensis*	0.99952	1.02 ± 0.00	1.73 ± 0.05
12	AN-12	*Streptomyces fulvissimus*	0.99996	1.95 ± 0.20	2.45 ± 0.20
13	AN-13	*Streptomyces lydicus*	0.83566	1.66 ± 0.02	1.96 ± 0.00
14	AN-14	*Streptomyces lydicus*	0.96220	1.37 ± 0.01	1.69 ± 0.02
15	AN-15	*Streptomyces purpureus*	0.99887	1.43 ± 0.04	1.83 ± 0.03
16	AN-16	*Streptomyces lydicus*	0.98637	1.28 ± 0.06	1.69 ± 0.01
17	AN-17	*Streptomyces griseoviridis*	0.98475	1.22 ± 0.03	1.83 ± 0.06
18	AN-18	*Streptomyces longisporoflavus*	0.98250	1.10 ± 0.04	1.73 ± 0.05
19	AN-19	*Streptomyces xanthochromogenes*	0.99766	1.16 ± 0.03	1.58 ± 0.02
20	AN-20	*Streptoverticillium olivoverticillatum*	0.99270	1.74 ± 0.05	2.12 ± 0.05
21	AN-21	*Streptomyces longisporoflavus*	0.98250	1.08 ± 0.00	1.93 ± 0.03
22	AN-22	*Streptomyces nogalater*	0.99996	1.53 ± 0.02	1.85 ± 0.02
23	AN-23	*Streptoverticillium olivoverticillatum*	1.00000	1.16 ± 0.01	1.68 ± 0.02
24	AN-24	*Streptomyces nogalater*	0.99295	1.88 ± 0.23	2.61 ± 0.23
25	AN-25	*Streptoverticillium olivoverticillatum*	0.99978	1.22 ± 0.01	1.70 ± 0.04
26	AN-26	*Streptomyces pactum*	0.99996	1.35 ± 0.03	1.84 ± 0.02
27	AN-27	*Streptomyces longisporoflavus*	0.97547	1.65 ± 0.15	2.42 ± 0.15
28	AN-28	*Streptomyces xanthochromogenes*	0.99881	1.25 ± 0.04	1.25 ± 0.01
29	AN-29	*Streptomyces aureofaciens*	0.91253	1.31 ± 0.02	1.31 ± 0.02
30	AN-30	*Streptomyces chattanoogensis*	0.88438	1.40 ± 0.00	1.40 ± 0.01
31	AN-31	*Streptomyces cellulosae*	0.97560	2.21 ± 0.03	2.21 ± 0.03
32	AN-32	*Streptoverticillium olivoverticillatum*	0.99978	1.45 ± 0.01	1.45 ± 0.03

The phosphatise-solubilising activity index is the halo ratio of clear zone / by colony size of tested actinomycete on Pikovskaya medium. Data are mean ± SD (n ≥ 3). PIBWIN ID score is probability calculated by the software PIBWIN.

**Table 2.** Enzyme activity indices thermo-tolerant actinomycetes isolates after five days of incubation

**Isolates**	**Cellulase**	**Chitinase**	**Pectinase**	**Protease**	**Lipase**	**Amylase**
*S. fulvissimus* AN-12						
28°C	1.38 ± 0.01	1.50 ± 0.02	1.30 ± 0.01	1.32 ± 0.03	1.32 ± 0.02	1.31 ± 0.02
50°C	1.41 ± 0.03	1.58 ± 0.04	1.41 ± 0.00	1.36 ± 0.03	1.33 ± 0.01	1.29 ± 0.03
*S. olivoverticillatum* AN-20						
28°C	1.42 ± 0.05	1.31 ± 0.05	1.35 ± 0.00	1.25 ± 0.03	1.34 ± 0.02	1.33 ± 0.04
50°C	1.59 ± 0.01	1.22 ± 0.07	1.32 ± 0.00	1.40 ± 0.03	1.37 ± 0.01	1.39 ± 0.05
*S. nogalater* AN-24						
28°C	1.36 ± 0.03	1.32 ± 0.05	1.35 ± 0.02	1.34 ± 0.03	1.22 ± 0.00	1.35 ± 0.01
50°C	1.66 ± 0.02	1.42 ± 0.03	1.42 ± 0.01	1.35 ± 0.03	1.23 ± 0.01	1.38 ± 0.00
*S. longisporoflavus* AN-27						
28°C	1.42 ± 0.02	1.35 ± 0.04	1.40 ± 0.03	1.46 ± 0.03	1.27 ± 0.02	1.34 ± 0.03
50°C	1.36 ± 0.04	1.39 ± 0.03	1.42 ± 0.01	1.39 ± 0.03	1.30 ± 0.04	1.29 ± 0.04
*S. cellulosae* AN-31						
28°C	1.30 ± 0.04	1.24 ± 0.04	1.35 ± 0.05	1.33 ± 0.03	1.33 ± 0.02	1.32 ± 0.05
50°C	1.49 ± 0.01	1.38 ± 0.05	1.38 ± 0.01	1.34 ± 0.03	1.34 ± 0.04	1.34 ± 0.02

The enzyme index is the halo ratio of clear zone / colony size of tested actinomycete isolate. Data are means ± SD (n ≥ 3).

### Identification of phosphate solubilizing actinomycetes.

All of the 32 actinomycete isolates were identified with the use of PIBWIN software (15). Actinomycetes isolates identified were mainly *Streptomyces* (28 isolates) and secondly *Streptoverticillium* genus (04 isolates) show in Table 1. Majority of the actinomycetes identified had identification score above 0.95 which is the threshold ID score. All of the 32 isolates were tested for ability to solubilize phosphate at 28°C and 50°C. The actinomycetes *S. fulvissimus* AN-12, *S. olivoverticillatum* AN-20, *S. nogalater* AN-24, *S. longisporoflavus* AN-27 and *S. cellulosae* AN-31 were selected for consortium and were studied further for phosphate solubilization.

### Enzyme activity indices of thermo-tolerant actinomycetes.

To prepare multifunctional biofertilizer thermo-tolerant phosphate solubilizing actinomycetes selected were *S. fulvissimus* AN-12, *Streptoverticillium olivoverticillatum* AN-20, *S. nogalater* AN-24, *S. longisporoflavus* AN-27 and *S. cellulosae* AN-31 grew well at higher temperature of above 50°C. Purpose of selection of these five actinomycetes was because these have other biochemical activities useful for composting and for biofertilizer. These enzyme activities studies were cellulase, chitinase, pectinase, lipase and amylase required for humus formation and decomposition of cellulose, pectin, chitin, lipid and carbohydrates in addition to phosphate solubilization. These five actinomycetes are good producer of these enzymes required for bio-fertilizer.

The actinomycetes *S. fulvissimus* AN-12, *S. olivoverticillatum* AN-20, *S. nogalater* AN-24, *S. longisporoflavus* AN-27 and *S. cellulosae* AN-31 were selected and studied for phosphate solubilization at temperature 28°C and 50°C on the medium Pikovskaya’s broth and Rock Phosphate broth.

In Pikovskaya’s broth maximum soluble phosphate was released by *S. longisporoflavus* AN-27 (181.25 μg ml^−1^) at 28°C followed by *S. fulvissimus* AN-12 (135.43 μg ml^−1^), and *S. olivoverticillatum* AN-20 (125.53 25 μg ml^−1^). Relatively low amount of phosphate was solubilized by *S. cellulosae* AN-31 (89.78 μg ml^−1^) and *S. nogalater* (68.66 μg ml^−1^). At 50°C phosphate solubilization was highest for *S. olivoverticillatum* AN-20 (176.67 μg ml^−1^). The phosphate solubilization in Rock phosphate broth at 28°C and 50°C was maximum for *S. longisporoflavus* AN-27 was 190.36 μg ml^−1^ and for *S. olivoverticillatum* AN-20 was 180.87 μg ml^−1^ shown in Table 3.

**Table 3.** Phosphate solubilising activity and pH of tested thermo-tolerant phosphate solubilising actinomycete isolates.

**Actinomycete Isolates**	**Pikovskaya’s broth**		**Rock Phosphate broth**	
	
	Soluble P (μg ml^−1^)	pH	Soluble P (μg ml^−1^)	pH
*S. fulvissimus* AN-12				
28°C	135.43 ± 0.01	6.7 ± 0.1	125.55 ± 0.02	6.7 ± 0.2
50°C	152.86 ± 0.03	6.7 ± 0.0	144.59 ± 0.04	6.7 ± 0.1
*S. olivoverticillatum* AN-20				
28°C	125.53 ± 0.05	6.7 ± 0.2	133.81 ± 0.05	6.7 ± 0.1
50°C	176.67 ± 0.01	6.7 ± 0.0	180.87 ± 0.07	6.9 ± 0.1
*S. nogalater* AN-24				
28°C	58.66 ± 0.03	6.6 ± 0.1	78.01 ± 0.05	7.0 ± 0.2
50°C	102.69 ± 0.02	6.8 ± 0.1	109.62 ± 0.03	6.8 ± 0.1
*S. longisporoflavus* AN-27				
28°C	191.25 ± 0.02	6.4 ± 0.0	190.36 ± 0.04	6.7 ± 0.0
50°C	172.72 ± 0.04	6.9 ± 0.1	150.57 ± 0.03	6.7 ± 0.1
*S. cellulosae* AN-31				
28°C	89.78 ± 0.04	7.1 ± 0.0	94.24 ± 0.04	6.5 ± 0.1
50°C	155.02 ± 0.01	6.6 ± 0.1	145.33 ± 0.05	6.4 ± 0.0

Data are the means ± SD (n ≥ 3).

The amount of phosphate released in the Pikovskaya’s broth by each of the five actinomycete isolates was quantitatively measured using chlorostannous reduced molybdophosphoric acid blue method describe by Jackson (27). In quantitative estimation, range of tricalcium phosphate solubilization was found to range between 58.66 to 191.25 μg ml^−1^ shown in Table 3.

### Biofertilizer preparation.

**Fig. 1.**
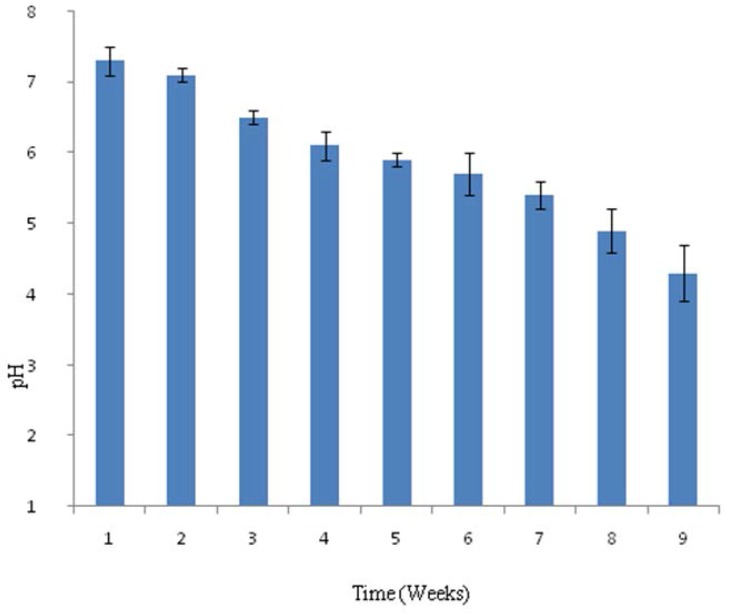
pH of the Biofertilizer prepared of actinomycetes consortium. Changes in the pH during biofertilizer preparation were recorded at the end of every week from 1^st^ week to 8^th^ week. The bars on the histogram denote standard deviation from three rounds of analysis.

**Fig. 2.**
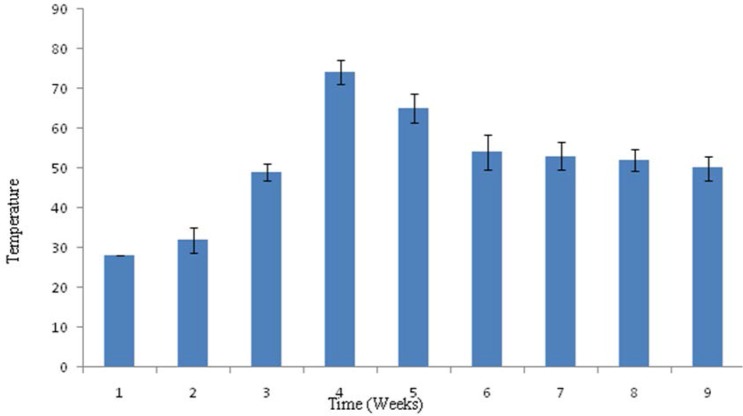
Temperature record of Biofertilizer prepared with Actinomycetes consortium. Changes in temperature during biofertilizer preparation were measured at the end of every week from 1^st^ week to 8^th^ week. The bars on the histogram denote standard deviation from three rounds of analysis.

**Fig. 3.**
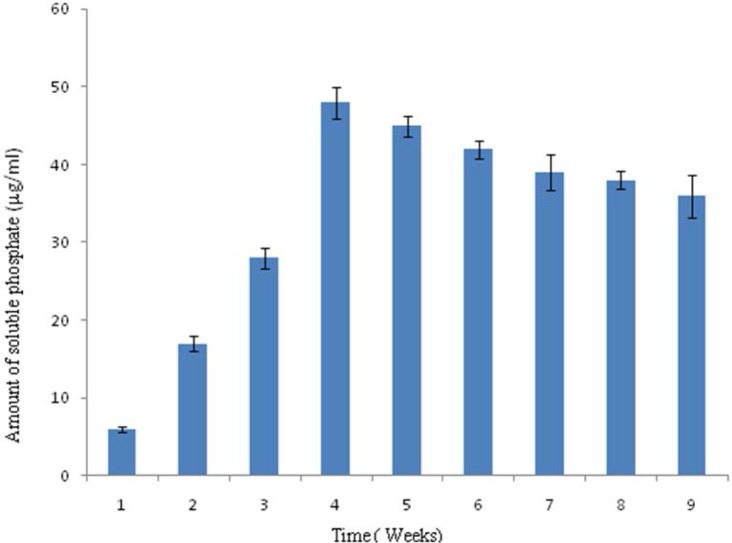
Soulubilized Phosphate generated in μg ml^−1^ . Amounts of solubilized phosphate generated during biofertilizer preparation were measured at the end of every week from 1^st^ week to 8^th^ week. The bars on the histogram denote standard deviation from three rounds of analysis.

Composting was done in 100 L capacity drum and raw material of compost was inoculated with 1 L of broth grown with five actinomycete consortium (10^7^ spores ml^−1^). Parameters; Temperature, pH and amount of soluble phosphate released were measured at the end of week for up to eight weeks. The pH recorded in the composting process showed that there was decline in the pH on composting during biofertilizer preparation. The pH at the beginning was slightly alkaline, 7.5 and there was continuous decline observed from 2^nd^ week to 8^th^ week of composting. The pH of composting on 8^th^ week was pH 4.9 (Fig. 1). The temperature was around 30°C at the beginning of the bio-fertilizer preparation. On the fourth week it reached to maxima of 76°C and decline of the temperature was observed from 4^th^ week to 8th weeks of the composting, shown in Fig. 2. The solubilized phosphate was recorded from the 1^st^ week to 8^th^ week, which was found to be 6 μg ml^−1^ at the beginning and 48 μg ml^−1^ in the 4^th^ week. The solubilised phosphate was found to decline after 4^th^ week, which was found to be 36 μg ml^−1^ in the 8^th^ week, shown in Fig 3. The consortium of actinomycetes *S. fulvissimus* AN-12, *S. olivoverticillatum* AN-20, *S. nogalater* AN-24, *S. longisporoflavus* AN-27 and *S. cellulosae* AN-31 showed excellent potential to solubilize phosphate in the composting.

## DISCUSSION

In the study we have isolated, identified and developed an actinomycetes consortium inoculum from five thermotolerant actinomycetes. We report production of biofertilizer that have several essential biocatalytic activity and prominently phosphate solubilization by keeping soilt health at the centre. There seems to have an advantage of selecting thermo-tolerant actinomycete as they can solubilize phosphate at composting temperature which is usually high. Mesophilic inoculants wouldn’t survive this temperature. The best part would be when applied this fertilizer in the field the actinomycetes of this consortia would still carry on their decomposing activities.

Screening of rock phosphate solubilizing actinomycetes was successfully explored by Hamdali et al. (29). Actinomycetes studied for phosphate solubilizing potential belong to the genus *Micromonospora*. In the biofertilizer preparation from poultry waste (30, 31) used phosphate solubilizing actinomycete *Streptosporangium* spp from earthworm casts of *Eudrilus eugeniae* with cellulolytic potential. The *Streptosporangium* spp was found to be cellulytic, acidogenic, acid tolerant and phosphate solubilizing.

Since Actinomycetes are able to produce spores, a form of dissemination and sustenance during adverse conditions (32, 33), these bacteria could be used for the formulation of novel biofertilizer and bio-control products constituted by spores and/or mycelium of the adhoc Actinobacteria in association with pulverized rock phosphate. Attempts are being made to prepare phosphate solubilizing biofertilizers by using yeast strains capable of solubilizing phosphate. Yeasts used were *Rhodotorula* sp., *Candida rugosa*, *Saccharomyces cerevisiae* and *Saccharomyces rouxii*. Filamentous fungi were also used as biofertilizer and for phosphate solubilisation by *Penicillium expansum*, *Mucor ramosissimus* and *Candida krissii*, from phosphate mines (34).

Many strains of bacteria have been identified as PSB, including *Pseudomonas putida*, *Microbacterium laevaniformans* and *Pantoea agglomerans*, those are highly efficient converting insoluble phosphate to solubilised phosphate. Recently, researchers at Colorado State University demonstrated that a consortium of four bacteria synergistically solubilize phosphorus at a much faster rate than any single strain alone (35). This study demonstrated that the thermo-tolerant actinomycetes screened with multifunctional phosphate solubilizing property as do other soil borne microorganisms such as *Pseudomonas putida* (36), *Acinetobacter* sp and *Serratia* species (37), that were able to solubilize insoluble phosphate as well as produce IAA, chitinase, β-1,3-glucanase, siderophores and anti-fungal substances to improve plant growth. Commercial products containing *Streptomyces griseoviridis* (Mycostop®) and *Streptomyces lydicus* (e,g, Actinovate® and Actino-Iron®) were applied to control important plant pathogens such as *Fusarium oxysporum, Pythium ultimum, Botrytis cinera* and *Alternaria brassicicola* (38–40).

The use of proficient phosphate solubilizing microorganisms opens up a new prospect for better crop productivity besides improving soil health. However, the feasibility and sustainability of PSM technology mainly depends on the development and delivery of good quality inoculants to farmers. Therefore, there is a need for extensive and consistent research efforts to identify and characterise more PSM with greater efficiency for their ultimate application under field conditions. Microbiologists have a great responsibility towards the society to find ways and means as to how soil phosphate could be improved without applying the chemical phosphate fertilizers under different agro-climatic regions of the world. The present bio-fertilizer inoculum could be applied for composting of the large available raw waste material like crop waste, livestock waste, human habitation wastes, by-product of agricultural industries, water hyacinth and weeds etc (9).

The promise of exploiting soil microorganisms to increase mobilization of soil phosphate remains to be explored to the fullest. This will be achieved through better management of soil microbial communities, by development of more effective microbial inoculants, though the genetic manipulation of specific organisms or with combination of these approaches is not known. What is clear though is that soil microorganisms play an important role in the mobilization of soil phosphate and the detailed understanding of their contribution to the cycling of phosphate in soil plant systems is required for the development of sustainable agriculture and our movement from a green revolution to an evergreen revolution can be accomplished. The reported actinomycetes consortium inoculum in present study holds a great significance as it could withstand high temperatures and solubilize insoluble phosphate to a great extent. This work therefore is expected to contribute to the development of sustainable agriculture cleaner than the present one.
